# Emerging insights into the gut microbiota as a key regulator of immunity and response to immunotherapy in hepatocellular carcinoma

**DOI:** 10.3389/fimmu.2025.1526967

**Published:** 2025-02-25

**Authors:** Siqi Ren, Yinping Zhang, Xingyue Wang, Jiahong Su, Xiang Wang, Zijun Yuan, Xinyu He, Sipeng Guo, Yu Chen, Shuai Deng, Xu Wu, Mingxing Li, Fukuan Du, Yueshui Zhao, Jing Shen, Wei Hu, Xiaobing Li, Zhangang Xiao

**Affiliations:** ^1^ Laboratory of Molecular Pharmacology, Department of Pharmacology, School of Pharmacy, Southwest Medical University, Luzhou, China; ^2^ Research and Experiment Center, Sichuan College of Traditional Chinese Medicine, Mianyang, China; ^3^ Cell Therapy and Cell Drugs of Luzhou Key Laboratory, Luzhou, China; ^4^ South Sichuan Institute of Translational Medicine, Luzhou, China; ^5^ Department of Gastroenterology, Shenzhen Hospital, Southern Medical University, Shenzhen, Guangdong, China; ^6^ Gulin Traditional Chinese Medicine Hospital, Luzhou, China

**Keywords:** gut microbiota, hepatocellular carcinoma (HCC), tumor microenvironment (TME), antibiotics, probiotics, prebiotics, fecal microbiota transplantation (FMT), immunotherapy

## Abstract

The gut microbiota, a complex microbial ecosystem closely connected to the liver via the portal vein, has emerged as a critical regulator of liver health and disease. Numerous studies have underscored its role in the onset and progression of liver disorders, including alcoholic liver disease, metabolic dysfunction-associated steatotic liver disease (MASLD), metabolic dysfunction-associated steatohepatitis (MASH), liver fibrosis, cirrhosis, and hepatocellular carcinoma (HCC). This review provides a comprehensive overview of current insights into the influence of the gut microbiota on HCC progression, particularly its effects on immune cells within the HCC tumor microenvironment (TME). Furthermore, we explore the potential of gut microbiota-targeted interventions, such as antibiotics, probiotics, prebiotics, and fecal microbiota transplantation (FMT), to modulate the immune response and improve outcomes of immunotherapy in HCC. By synthesizing insights from recent studies, this review aims to highlight microbiota-based strategies that may enhance immunotherapy outcomes, advancing personalized approaches in HCC treatment.

## Graphical Abstract

In the progression from normal liver to MASLD/alcoholic fatty liver, MASH/alcoholic steatohepatitis, cirrhosis/liver fibrosis, and HCC, the gut-liver axis plays a key role. The metabolic products of the gut microbiota influence the liver via the portal vein, which in turn impacts HCC tumor cells, immune cells in the TME, and even the therapeutic efficacy of ICIs. Various intervention strategies, including antibiotics, probiotics, prebiotics, bacteriophage, FMT, traditional Chinese medicine preparations, and diet, have the potential to improve gut microbiota. Current research suggests that antibiotics can influence the efficacy of ICIs in HCC by modulating the gut microbiota, while probiotics, bacteriophage therapy, prebiotics, and FMT may optimize gut microbiota composition to enhance ICIs therapy in the future. Additionally, interventions like antibiotics, probiotics, FMT, and traditional Chinese medicine preparations can modulate immune cells within the HCC TME by improving gut microbiota balance. Bacteriophage therapy, prebiotics, and dietary modifications also hold promise for future regulation of immune cells in the TME via gut microbiota modulation. MASLD, metabolic dysfunction-associated steatotic liver disease; MASH, metabolic dysfunction-associated steatohepatitis; HCC, hepatocellular carcinoma; FMT, fecal microbiota transplantation; TME, tumor microenvironment; ICIs, immune Checkpoint Inhibitors.

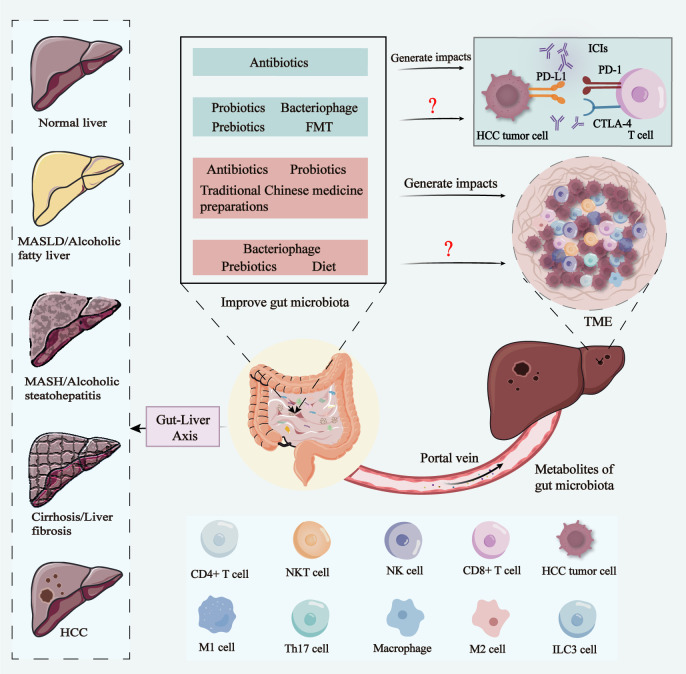

## 1 Introduction

### 1.1 Gut microbiota

The gut microbiota is a highly intricate community in the gastrointestinal tract of humans that comprises approximately 10 to 100 trillion microorganisms (1, 2), encompassing fungi, bacteria, viruses, and parasites (3). The collective genome of the human gastrointestinal tract is known as the gastrointestinal microbiome (4). While the human genome is estimated to consist of approximately 23,000 coding genes, the gastrointestinal microbiome harbors over 3 million coding genes. These genes have the capacity to generate thousands of metabolites that influence the host’s health and disease progression through intricate biological interactions (5). The primary metabolites produced by the gut microbiota consist of short-chain fatty acids (SCFAs), tryptophan, bile acids (BAs), lipopolysaccharides (LPS), and indole derivatives (6–10). The gut microbiota plays a crucial role in the human body, not only pertaining to intestinal health but also exerting influence on the pathogenesis and progression of various diseases as well as immune system development (11–14).

### 1.2 Hepatocellular carcinoma

Liver cancer is classified into primary and secondary types. Primary liver cancer is a highly malignant tumor that accounts for the majority of cases. HCC is the most common primary liver cancer, accounting for approximately 90% of cases (15). There are a number of staging systems for HCC that comprehensively assess the size, number, and aggressiveness of the tumor, as well as the overall health of the patient, in order to better guide treatment and assess prognosis. For example, the American Association for the Study of Liver Diseases (AASLD) guidelines, the World Health Organization (WHO) staging system, and the Barcelona Clinical Liver Cancer Staging (BCLC) system, which is recognized as one of the most effective staging methods due to its accuracy and practicality (16). In the BCLC staging system, early HCC refers to stages 0 and A, while middle and late HCC correspond to stages B, C and D. Early HCC can be completely cured by radical means such as hepatectomy and liver transplantation, but about 70% of patients have progressed to the middle and late stages at the time of diagnosis and are unable to undergo surgery (16). Therefore, non-surgical treatment has become the main treatment method for patients with advanced HCC. Non-surgical treatment is divided into local (interventional, ablative, radiotherapy) and systemic therapy (molecular targeting, immunotherapy). Among them, immune checkpoint inhibitors (ICIs) are the focus of current HCC immunotherapy research. They can not only block the immune escape of tumor cells, but also activate the function of T cells and enhance the immune surveillance and killing ability of T cells to generate immune response (17). Common immune checkpoints include PD-1, PD-L1, and cytotoxic T lymphocyte-associated protein 4 (CTLA-4). Currently PD-1/PD-L1 inhibitors approved for the treatment of HCC include sindilizumab, karelizumab, atezolizumab, durvalumab, etc. CTLA-4 inhibitors include terelizumab, and these inhibitors have been shown to be effective in some patients with advanced HCC (18). In addition, a combination of atezolizumab and bevacizumab (Atez/Bev) is recommended as first-line treatment for patients with advanced HCC (19).

## 2 Gut microbiota and HCC

### 2.1 Effects of gut microbiota on liver health before HCC development

The main causes of HCC are viral infections, such as chronic hepatitis B (20) and hepatitis C (21) viral infections. However, the introduction of the hepatitis B vaccine has diminished the role of viral infection as the primary precursor of HCC (22). More than 90% of HCC cases currently occur in patients with cirrhosis (23), cirrhosis is widespread worldwide and can be caused by different causes, such as obesity, metabolic dysfunction-associated steatotic liver disease (MASLD), excessive alcohol consumption, hepatitis B or C infections, autoimmune diseases, cholestatic diseases, and iron or copper overload (24). Notably, a recent systematic review and meta-analysis clearly showed that the prevalence of sarcopenia in HCC patients was approximately 39%, and that sarcopenia was independently associated with decreased overall survival and progression free survival in HCC, independent of treatment modalities (25). On a related note, another systematic review and meta-analysis clearly indicated that the intestinal microbiota alpha diversity was significantly lower in patients with sarcopenia compared to non-sarcopenia groups (26). These findings underscore the complex interplay between metabolic health, muscle mass, and gut microbiota in HCC progression.

*Akkermansia muciniphila* (*A. muciniphila*) is a symbiotic bacterium residing in the intestinal mucosal layer that holds great potential for use as a probiotic (27). In a study on *A. muciniphila* and HCC, dysbiotic animals with HCC exhibited an intrahepatic immunosuppressive microenvironment characterized by intestinal barrier damage, bacterial translocation-induced monocyte myeloid-derived suppressor cell (MDSC) expansion, and inhibition of CD4+T and CD8+T cells, but they were reversed upon treatment of animals with *A. muciniphila*. Therefore, the absence of *A. muciniphila* in the intestine may contribute to the progression of liver diseases such as hepatitis and liver fibrosis toward HCC (28).

In 1992, the Food and Agriculture Organization of the United Nations defined resistant starch as “the collective term for starch and its degradation products that evade absorption by the small intestine in healthy individuals” (29). An experimental study using resistant starch as a dietary supplement suggests that alterations in the gut microbiome may mediate the progression of MASLD. Specifically, compared with the control group, the concentration of triglycerides in the liver of the resistant starch intervention group was significantly reduced (9.08%), the specific mechanism of which is that resistant starch reduces *Bacteroides stercoris*, the intestinal bacteroides that is significantly related to the content of triglycerides in the liver and liver enzymes (30).

Yujun et al. discovered that soluble fiber inulin was more effective than insoluble fiber cellulose in inhibiting the progression of metabolic dysfunction-associated steatohepatitis (MASH) in mice (31). It reduces hepatic steatosis, necrotizing inflammation, edema and fibrosis. The specific mechanism is that the use of inulin leads to the enrichment of distasonis in symbiotic parabacteroides, and these symbiotic parabacteroides are able to restore intestinal barrier function through the production of pentaconic acid by inulin, thereby reducing the expression of serum lipopolysaccharide and liver pro-inflammatory cytokines (31). Similarly, another group demonstrated that consumption of inulin caused alterations to the gut microbiota, characterized by enrichment in *Bacteroides* and an increase in concentrations of SCFAs, particularly acetate, within portal venous blood. Acetate inhibits the progression of MASLD or MASH through the regulation of liver lipid metabolism and affects insulin sensitivity via activation of free fatty acid receptor 2 in hepatic cells (32). In addition, we have summarized important clinical trials over the past 17 years on improving gut microbiota for the treatment of MASLD, MASH, alcohol associated chronic liver disease, and cirrhosis (**Table 1**).

**Table 1:** Important clinical trials on improving gut microbiota for the treatment of pre HCC related diseases.

Start time	Disease	Improvement methods	Research objective	Method	Phase	Clinical trial ID
2007	Cirrhosis	Probiotics	Determine the effectiveness of probiotics in preventing spontaneous bacterial peritonitis in patients with cirrhosis.	Primary prevention: Take probiotics (VSL#3) and placebo (capsules containing lactose powder and similar appearance) for one year or until the end of the study. Secondary prevention: Take probiotics and norfloxacin (400mg/day) for one year or until the end of the study.	II/III	NCT00678613
2009	MASH	Omega-3 fish oil	Determine the effect of supplementing Omega-3 fish oil on liver gene expression in MASH patients and evaluate its impact on the patient’s gut microbiota.	Omega-3 fatty acids in the form of fish oil capsules (2g/d).	II	NCT01056133
2015	MASH	FMT	Determine if FMT, using stool from lean donors, is an effective and safe treatment for MASH.	During upper gastrointestinal endoscopy, frozen feces from a lean healthy donor are injected into the duodenum through the working channel of the instrument, with a dose of 1 dose.	I	NCT02469272
2016	MASLD	FMT	To determine the impact of alterations of the gut microbiome on parameters of insulin resistance and on liver fat content.	Group 1: Patients will be randomized to receive fecal transplantation of their own microbes/feces (autologous-9 cases), the dosage: about 100ml. Group 2: Patients will be randomized to receive fecal transplantation of microbiome/stool from a healthy donor (allogeneic-12 cases), dosage: about 100ml.	I/II	NCT02496390
2016	Cirrhosis	FMT	Determine whether FMT is an effective and safe method for treating decompensated cirrhosis.	Experimental: Traditional treatment/FMT. control group: Traditional treatment.	I/II	NCT03014505
2017	Cirrhosis	FMT	To evaluate the safety and tolerability of oral fecal transplant in patients with cirrhosis and hepatic encephalopathy.	Experimental: Fifteen FMT Openbiome capsules administered at the same time. Control group: Fifteen placebo capsules administered at the same time.	I	NCT03152188
2018	Cirrhosis	FMT	Assessing the feasibility and safety of using FMT to stabilize gut microbiota dysbiosis in patients with advanced liver cirrhosis.	Experimental group: FMT (200ml) was injected into the duodenum through gastroscopy. Control group: A placebo solution with the same appearance (200ml of 0.9% saline and 12.5% glycerol) was injected into the duodenum through gastroscopy in a single blind manner.	III	NCT02862249
2019	MASLD	FMT	Study the effect of consecutive FMT on liver fat accumulation at 12 weeks.	Twenty patients were randomly assigned to receive intravenous infusion of allogeneic or autologous feces via gastroscopy in a 1:1 ratio at time points of weeks 0, 3, and 6.	IV	NCT04465032
2019	Cirrhosis	Synbiotics	Study on changes in gut microbiota and metabolic characteristics of liver cirrhosis patients supplemented with lactulose, *Clostridium butyricum*, and *Bifidobacterium infantis*.	Experimental: Orally administered Entecavir 0.5 mg, once a day. For six months. A 10-g packet of lactulose oral solution and three capsules of probiotics: Take the contents orally after meals three times a day. six months.	IV	NCT05687409
2019	Cirrhosis and hepatic encephalopathy	FMT	To evaluate the safety and tolerability of fecal transplant in patients with cirrhosis and hepatic encephalopathy.	Experimental: Dual Oral and rectal FMT at visit 2 Oral FMT at day 30. Control group1: Oral FMT and rectal placebo at visit 2 Oral FMT at day 30. Control group2: Oral placebo and rectal FMT at visit 2 Oral placebo at day 30. Placebo Comparator: Oral and rectal placebo at visit 2 Oral placebo at day 30.	I/II	NCT03796598
2022	Alcohol-associated chronic liver disease and cirrhosis	Intestinal microbiota transplant	To test the safety, tolerability, and effectiveness of the capsules that contain bacteria from healthy individuals when used to treat alcohol craving and drinking.	Experimental: Taking capsules containing freeze-dried gut microbiota from healthy human donors. Control group: Taking capsules containing non active substances (“sugar pills”).	I/II	NCT05548452
2022	MASH	FMT	Evaluate the effectiveness and safety of microbiota manipulation therapy for MASH patients through FMT.	Lean healthy donor frozen fecal microbiota will be administered via duodenal infusion in an upper gastrointestinal endoscopy.	I	NCT03803540
2022	MASLD	Prebiotics (inulin rich in oligofructose)	Study on the efficacy of soluble fiber supplements in the treatment of pediatric MASLD.	Experimental: The intervention group will receive a daily fiber supplementation of (fructo-oligosaccharide enriched inulin, 4g twice daily). Control group: Carbohydrate placebo supplement daily (4g dose twice daily).	I	NCT05480696
2023	MASLD	Diet fermented soybean extract (MBS-217)	Exploring the efficacy and safety of MBS217 in treating MASLD participants, as well as investigating changes in other metabolic parameters, gut microbiome, metabolomics, and intestinal permeability.	Experimental group: 4 ml MBS-217 twice a day for 16 weeks. Control group: 4 ml MBS-217 placebo twice a day for 16 weeks.	I	NCT05686174
2023	MASH	FMT; probiotics; prebiotics	To investigate the therapeutic potential of *A. soehngenii* and pasteurized *Bacillus mucophilus* in combination with animal *Bacillus lactic acid subspecies* and fructooligosaccharide in combination with or without conditioned vegetarian freeze-dried fecal microbiota transplantation capsules to reduce MASH in patients with fibrotic MASH.	Experimental: Oral freeze-dried FMT capsules, *A. soehngenii*, pasteurized *Bacillus mucophilus*, animal *Bacillus lactic acid subspecies* and fructooligosaccharide. Control group: Oral Placebo capsules, *A. soehngenii*, pasteurized *Bacillus mucophilus*, animal *Bacillus lactic acid subspecies* and fructooligosaccharide.	II	NCT05821010
2023	Cirrhosis	FMT	Investigating the beneficial effects of fecal transplantation on patients diagnosed with cirrhosis (regardless of the cause).	Experimental: The patient will undergo fecal transplantation using at least 70 grams of material collected and tested from healthy donors. Control group: Accept standard treatment.	III	NCT06478602
2023	Cirrhosis	FMT	Evaluate the efficacy and mechanism of encapsulated FMT (compared to placebo) in reducing infection and mortality in patients with alcohol-related and MASLD cirrhosis.	Experimental: Encapsulated FMT. Other: Placebo products containing microcrystalline methylcellulose.	III	NCT06461208

MASLD, metabolic dysfunction-associated steatotic liver disease; MASH, metabolic dysfunction-associated steatohepatitis; FMT, fecal microbiota transplantation.

### 2.2 Effects of gut microbiota metabolites on HCC

**Figure 1:**
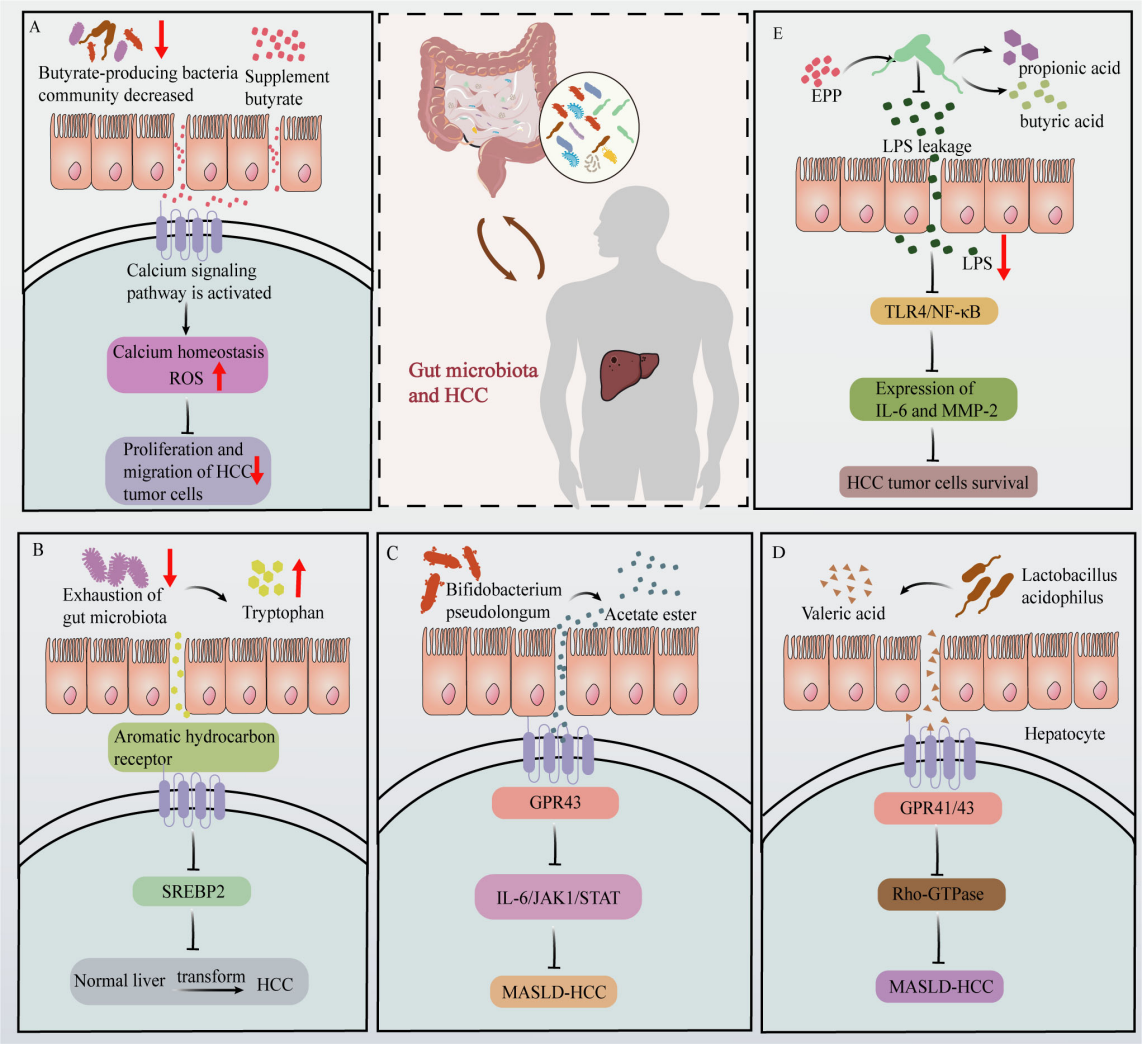
Effects of gut microbiota and their metabolites on HCC progression. **(A)** In HCC patients, the abundance of butyrate-producing bacteria is reduced. Butyrate supplementation activates calcium signaling pathways in HCC tumor cells, disrupting calcium homeostasis and increasing ROS production, ultimately inhibiting HCC tumor cells proliferation and migration. **(B)** Depletion of gut microbiota leads to increased tryptophan levels, which activate the aromatic hydrocarbon receptor. This activation suppresses SREBP2 expression, thereby inhibiting HCC development. **(C)** Acetate produced by Bifidobacterium pseudolongum binds to GPR43 receptors on HCC tumor cells, activating GPR43 and inhibiting the IL-6/JAK1/STAT3 signaling pathway, thus impeding MASLD-HCC progression. **(D)** The valeric acid produced by Lactobacillus acidophilus binds to the liver cell surface receptor GPR41/43, thereby exerting inhibitory effects on Rho-GTPase signaling and suppressing the development of MASLD-HCC. **(E)** EPP intervention increases the abundance of propionic and butyric acid-producing gut microbiota. This increase effectively inhibited the leakage of LPS, which subsequently led to the inhibition of the TLR4/NF-κB pathway of HCC tumor cells and further reduced the expression of IL-16 and MMP-2, ultimately destroying HCC tumor cells survival.

Dysregulation of the gut microbiota can result in increased intestinal permeability, thereby triggering microbial translocation and enhancing liver exposure to microbiota-derived products and metabolites that have an array of effects on the progression of HCC (33). The primary microbiota-derived SCFAs are acetic acid, propionic acid, butyric acid (34), and valeric acid (35). Studies have demonstrated that there is a reduction in the abundance of butyrate-producing bacteria in early-stage HCC. In addition, supplementation with butyrate can activates calcium-signaling pathways in HCC tumor cells, leading to dysregulation of calcium homeostasis and generation of reactive oxygen species, ultimately, this calcium-signaling cascade inhibits the proliferation and migration of HCC tumor cells (36) (**Figure 1A**). Additionally, the depletion of intestinal flora can hinder the metabolism of tryptophan in the intestine, leading to the accumulation of tryptophan and ultimately inhibiting the development of HCC (37). This process is mediated by the activation of aromatic hydrocarbon receptors by accumulated tryptophan and by inhibition of sterol regulatory element-binding protein 2 expression (**Figure 1B**).

Abnormal BAs metabolism is also closely associated with the progression of HCC and largely involves secondary BAs. The occurrence and development of HCC is a multifaceted process, and recent studies consistently indicate that the gut microbiota is significantly involved in modulating HCC signaling pathways. Acetate production by *Bifidobacterium pseudolongum* has been found to exert inhibitory effects on MASLD-associated HCC. Specifically, acetate is transported via the portal vein to the liver, where it selectively binds to GPR43 (G-protein coupled receptor 43) on HCC tumor cells. Activation of GPR43 suppresses the interleukin 6/Janus kinase 1/signal transducer and activator of transcription 3 signaling pathway, thereby impeding progression of MASLD-associated HCC (38) (**Figure 1C**). Research conducted by Yu Jun at the Chinese University of Hong Kong revealed that valeric acid produced by *Lactobacillus acidophilus* effectively reduced the mRNA and protein expression levels of markers associated with the Rho-GTPase (where Rho = Ras homology) pathway in mouse livers. Valeric acid was also found to inhibit Rho-GTPase signaling by binding to GPR41/43 receptors on hepatocyte surfaces, thereby suppressing MASLD-associated HCC (39) (**Figure 1D**).

Plant extracts can also affect HCC by modulating the production of metabolites within the gut microbiota. For instance, echinacea polysaccharide (EPP) possesses a diverse range of pharmacological activities, encompassing anti-inflammatory, immunomodulatory, and antineoplastic activities (40). Recent studies in mice have demonstrated the efficacy of EPP in attenuating hepatic damage induced by HCC, suppressing HCC proliferation, and inducing apoptosis. At a mechanistic level, EPP treatment caused a significant increase in the abundance of propionic acid- and butyric acid-producing gut microbes, such as *Coprococcus*, *Clostridium*, and *Roseburia*. This change in the gut microbiota promoted the expression of intestinal tight junction proteins and thus promoted intestinal repair, thereby inhibiting the leakage of the intestinal microbiota metabolite LPS. Consequently, in HCC tumor cells from mice, the Toll-like receptor 4/nuclear factor kappa-light-chain-enhancer of activated B cells (TLR4/NF-κB) pathway was inhibited, and the expression of inflammatory factors (e.g., interleukin 16 (IL-16)) and migration factors (e.g., matrix metalloproteinase-2) was reduced. Ultimately, these changes prevented the survival of HCC tumor cells (40) (**Figure 1E**).

It has also been shown that the gut microbiota can influence HCC through TLR4, and that hereditary TLR4 inactivation, intestinal sterilization, or sterility can reduce the incidence of HCC by approximately 80%. This reduction is primarily mediated by LPS (41).

### 2.3 Effects of gut microbiota on HCC immune cells

Various immune cells in the tumor microenvironment play an important role in tumor development, invasion and metastasis. The TME of HCC comprises cancer cells, immune cells, cancer-associated fibroblasts, cytokines, chemokines, and other components (42). Immune cell populations within the TME include T cells, B cells, dendritic cells (DCs), NK cells, M1 macrophages, M2 macrophages, and MDSCs (43). We comprehensively summarize the regulatory role of gut microbiota on immune cells in HCC TME and illustrate how optimizing gut microbiota affects immune cells in HCC TME.

#### 2.3.1 Effects of gut microbiota on T cells and NK cells in HCC

T lymphocytes are divided into two major distinct subsets, namely CD4+T cells (also known as T helper cells) and CD8+ T cells (also known as cytotoxic T-lymphocytes) (44). CD8+T cells play a pivotal role in the anti-tumor response by recognizing the major histocompatibility complex polypeptide complex expressed on cancer cells via their T cell receptors. CD4+T cells can be classified into two distinct subsets, namely Th1 and Th2 subsets (45). The Th1 subset exhibits direct cytotoxicity against cancer cells through the production of interferon gamma and tumor necrosis factor alpha (TNF-α). In contrast, the Th2 subset secretes anti-inflammatory mediators that facilitate tumor growth, such as IL-3 and IL-4 (46).

NKT cells are a distinct subset of T cells that possess both T cell receptors and NK cell receptors (47). NKT cells exhibit direct cytotoxicity against tumor cells via the Fas/FasL pathway, leading to the release of perforin, granzyme B, and TNF-α. Moreover, NKT cells can orchestrate the recruitment and activation of other immune cells by triggering the Th1 cytokine cascade, thereby indirectly exerting an anti-tumor effect (48).

NK cells were defined by Herberman in 1976 and named after their ability to autonomously kill target cells. NK cells play a crucial role in immune surveillance against tumors and, apart from exerting cytotoxic effects, secrete various cytokines, with INF-γ being the most prevalent (49). A recent Phase I clinical trial also demonstrated the advantageous role of NK cells in the treatment of HCC, with patients exhibiting a high objective response rate (63.6%) to hepatic arterial infusion chemotherapy combined with high-dose NK cell therapy (50).

**Figure 2:**
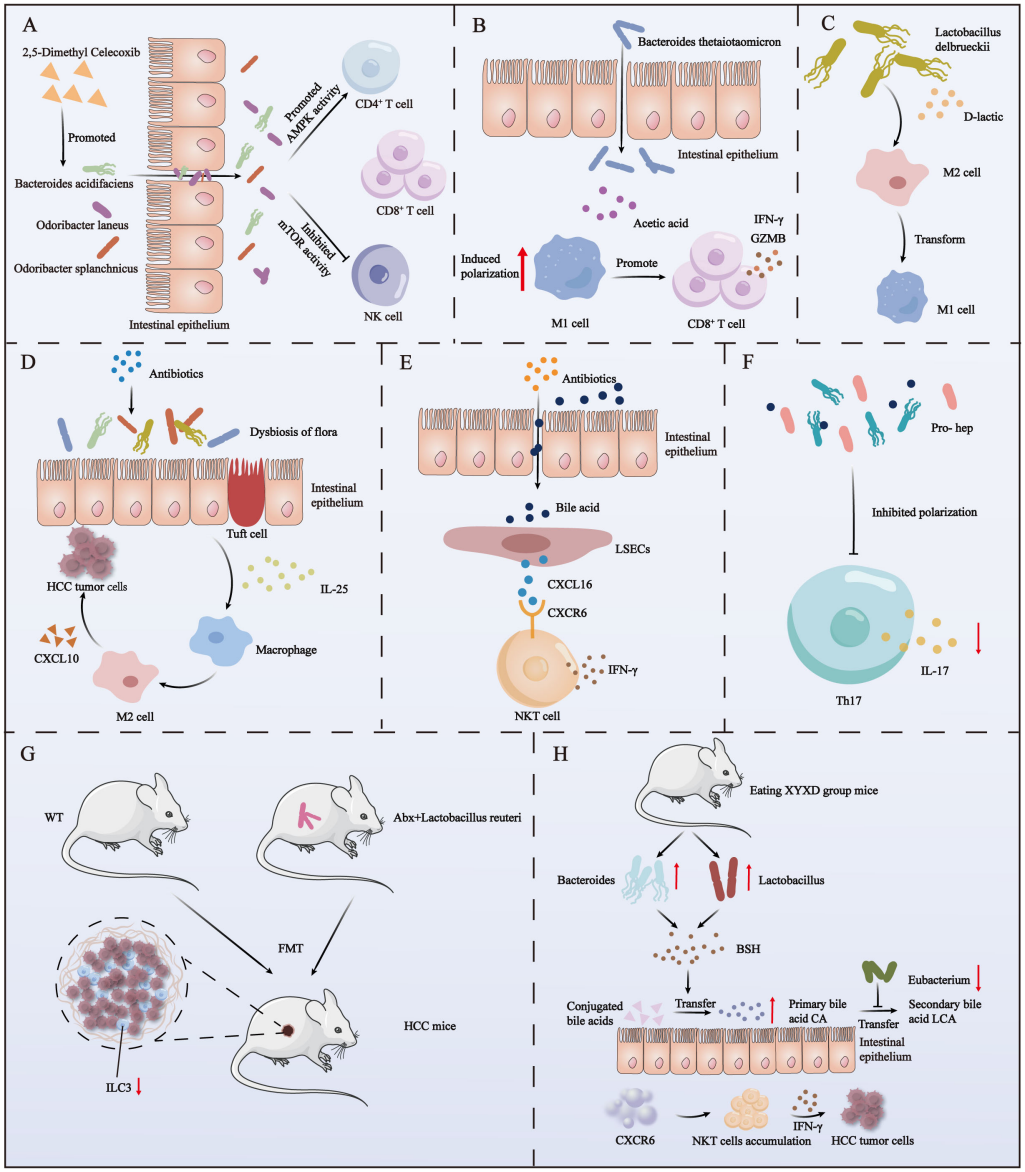
The impact of autogenous and post-intervention gut microbiota on immune cells in HCC. **(A)** The use of 2,5-dimethyl celecoxib activates three beneficial bacteria. These bacteria, in turn, activate the AMPK signaling pathway and inhibit the mTOR signaling pathway in NK, CD4^+^ T, and CD8^+^ T cells within HCC. **(B)** Acetate derived from *Bacteroides thetaiotaomicron* induces the polarization of M1 macrophages in HCC, enhancing the secretion of IFN-γ and GZMB by CD8^+^ T cells. **(C)** D-lactic acid, produced by the fermentation of Lactobacillus delbrueckii, converts M2 macrophages into M1 macrophages in HCC. **(D)** The use of antibiotics causes the imbalance of intestinal flora, which induces the proliferation of colon epithelial cluster cells to secrete a large amount of IL-25 and further promotes the vicarious activation of macrophages and the secretion of CXCL10, ultimately leading to the progression of HCC. **(E)** Antibiotic-induced dysbiosis promotes the secretion of the chemokine CXCL16 by liver sinusoidal endothelial cells via bile acids as messengers. CXCL16 acts on CXCR6^+^ liver NKT cells to produce IFN-γ. **(F)** The use of the probiotic mixture Pro-hep reduces Th17 cell polarization in HCC, subsequently downregulating IL-17 production. **(G)** Feces from WT mice or mice treated with antibiotics and then supplemented with Lactobacillus reuteri were transplanted into HCC mice, and a decrease in ILC3 cells in TME was found. **(H)** Mice with XYXD diet showed an increase in Bacteroides and Lactobacillus, a decrease in Eubacterium, and an increase in BSH (bile salt hydrolase) production. BSH converts conjugated bile acids into primary bile acids, while Eubacteria inhibits the conversion of primary bile acids to secondary bile acids. The accumulated primary bile acids ultimately trigger CXCR6’s recruitment of NKT cells in the liver and secretion of IFN-γ to combat HCC tumor cells.

The United States Food and Drug Administration has approved sorafenib as an anticancer medication for the management of unresectable HCC and advanced renal cell carcinoma (51). The anti-inflammatory drug 2,5-dimethylcelecoxib has been shown to synergize with sorafenib to enhance the sensitivity of HCC to sorafenib and inhibit the proliferation of HCC. Additionally, 2,5-dimethylcelecoxib promotes apoptosis (52). Recently, Pan et al. demonstrated that 2,5-dimethylcelecoxib can also affect the TME of HCC by modulating the gut microbiota to increase the abundance of beneficial bacteria. Specifically, 2,5-dimethylcelecoxib activates *Bacteroides acidifaciens*, *Odoribacter laneus*, and *Odoribacter* sp*lanchnicus*, which stimulate the adenosine monophosphate-activated protein kinase (AMPK) signaling pathway and inhibit the mammalian target of rapamycin (mTOR) signaling pathway in CD4+T/CD8+T and NK cells. These changes lead to an increase in the secretion of interferon gamma (IFN-γ) in CD4+T/CD8+T and NK cells and suppress the expression of programmed cell death protein 1 (PD-1) receptors. ultimately exerting a favorable influence on HCC (**Figure 2A**) (53).

#### 2.3.2 Effects of gut microbiota on other immune cells in HCC

In addition to T cells and NK cells in HCC TME, DCs, macrophages, B cells and MDSCs also play key roles in the development and immune response of HCC. The expression of BAs receptors has been observed in various immune cells, and the dysregulation or imbalance of BAs impacts the differentiation and function of diverse immune cells, thereby influencing the occurrence and progression of HCC (54).

DCs are a distinct subset of immune cells that can support innate immunity and elicit adaptive immunity (55, 56). The utilization of DC vaccine-based HCC therapy has emerged as a fundamental approach in personalized immunotherapy (57). A Phase II clinical trial demonstrated that a DC vaccine was well tolerated and safe in patients with HCC. Furthermore, the 6-month and 1-year post-treatment survival rates for 35 patients with advanced HCC were 33% and 11%, respectively (58).

Macrophages are classified into two categories based on their biological functions: M1 macrophages, which are classically activated; and M2 macrophages, which are alternatively activated (59). M1 macrophages secrete large amounts of various pro-inflammatory cytokines, such as TNF-α, IL-1β and inducible nitric oxide synthase, which have powerful pro-inflammatory and anti-tumor effects (60). The presence of M2 macrophages in the TME is associated with both inhibition of inflammation and promotion of tumor progression (61). Therefore, the balance of M1 and M2 macrophages in the TME is closely related to tumor progression.

MDSCs promote tumor progression via various mechanisms, such as by enhancing tumor cell survival, facilitating angiogenesis, and promoting invasion of and metastasis to healthy tissues (62). Additionally, MDSCs can have a dual effect on other immune cells through multiple mechanisms.

*Bacteroides* are predominant intestinal symbionts and thus play a crucial role in the degradation of complex polysaccharides and the maturation of the host immune system (63, 64). For example, *Bacteroides thetaiotaomicron* is a predominant constituent of the adult intestinal tract and is responsible for the degradation of plant polysaccharides, shaping the integrity of the intestinal mucosal barrier, promoting microecosystem stability, and conferring resistance against inflammatory changes (65). Moreover, this bacterium has been recognized as a key modulator of the gut microbiota ecosystem. Ma et al. identified a potential association between a decreased abundance of *B. thetaiotaomicron* and HCC relapse. Specifically, they found that acetic acid derived from *B. thetaiotaomicron* can induce M1 macrophage polarization in HCC, thereby increasing the secretion of CD8+T cells, IFN-γ, and granzyme B by cytokines and ultimately enhancing T cell-mediated tumor cell killing (**Figure 2B**) (66).

The endogenous immunomodulator d-lactic acid is produced through fermentation by *Lactobacillus delbrueckii* (67). D-lactic acid was found to induce the transformation of M2 macrophages into M1 macrophages in the HCC TME, thereby reshaping the immunosuppressive environment of HCC. It was determined that this transformation primarily involves D-lactic acid interacting with TLR2 and/or TLR9 receptors on macrophages, thereby inhibiting the phosphatidylinositol 3-kinase/Akt pathway and activating the NF-κB pathway. Ultimately, this process promotes the abovementioned transformation of M2 macrophages into M1 macrophages (68) (**Figure 2C**). This regulation of macrophage phenotypes by D-lactic acid is expected to improve the clinical outcome of tumor therapy. Therefore, regulating the metabolism of *L. delbrueckii* in the gut microbiota, especially its production of D-lactic acid, may become an important strategy for the treatment of HCC tumors.

## 3 The impact of the gut microbiota on HCC immunotherapy and the effects of improving the gut microbiota on HCC immune cells

Immunotherapies for HCC include ICIs, tumor vaccines, and chimeric antigen T-lymphocytes (69). There is a limited body of research on the relationship between gut microbiota and tumor vaccines and chimeric antigen T-lymphocyte therapy. Therefore, this discussion primarily focuses on ICIs immunotherapy. ICIs that target the programmed cell death pathway (PD-1/L1 and CTLA-4 are currently a major HCC treatment modality and constitute the largest proportion of immunotherapeutic approaches for this type of cancer (70). However, ICIs are not efficacious in all patients, approximately 60% of patients with HCC fail to respond to ICIs treatment, with only approximately 10%–20% being suitable to receive first-line ICIs therapy.

There are differences in the composition of gut microbiota between individuals who respond to ICIs and those who do not (71). For example, one study found that ICIs responders had a higher abundance of *Lachnoclostridium* and a higher ursodeoxycholic acid concentration than non-responders, but a lower abundance of *Prevotella_9*. In addition, patients with cancer often undergo conventional treatments such as chemotherapy and radiation prior to receiving immunotherapy, which may exert detrimental effects on the intestinal symbiosis system (72). Therefore, adjusting the gut microbiota to an optimal biodiversity and characteristic state prior to immune-related interventions may also be a new and effective treatment. A Phase II clinical trial is also designed to evaluate the efficacy and safety of oral enterobacter capsules in patients with advanced HCC who have progressed after immune checkpoint inhibitors combined with targeted anti-angiogenic drugs (NCT06563947).

In addition, research on the gut microbiota has shown that it has a profound impact on immune cells within the HCC TME. Therefore, improving the health of the gut microbiota to regulate the development of HCC has become a key strategy. These intervention measures include antibiotics, probiotics, fecal microbiota transplantation (FMT), prebiotics, dietary modifications, traditional Chinese medicine preparations, bacteriophages, etc.

### 3.1 Antibiotics

Antibiotics are a class of drugs that can inhibit the growth of or kill microorganisms and are widely used in the treatment of various infectious diseases (73–75). In recent years, there has been a growing focus on the correlation between antibiotics and cancer, and antibiotics have been identified as potentially supporting cancer treatment (76). However, the use of antibiotics to enhance the gut microbiota and consequently improve HCC immunotherapy has not been clinically documented. However, retrospective studies have indicated that antibiotics may affect HCC immunotherapy. One study encompassed all patients with advanced HCC who received ICIs treatment (including nivolumab, pembrolizumab, or ipilimumab) between January 2014 and December 2019, the findings revealed that antibiotic administration within a 30-day period before and after commencing ICIs treatment was associated with increased cancer-related mortality and overall mortality (77).

Relatedly, antibiotic usage was found to affect the efficacy of atezolizumab in combination with bevacizumab (denoted as “T + A therapy”) as a first-line standard treatment for unresectable HCC. Specifically, patients who received T + A therapy without antibiotics had a significantly longer progression-free survival and overall survival than their counterparts who received T + A therapy with antibiotics (78). However, some studies have suggested that patients with HCC may benefit from ICIs and antibiotic treatment. For example, a higher objective response rate was observed among patients with HCC who received early antibiotic treatment (within 30 days before or after ICIs treatment). However, the underlying mechanism was not elucidated (79).

In addition to affecting immunotherapy, the use of antibiotics also impacts the gut microbiome, thereby influencing tumor size in HCC mouse models. Moreover, antibiotics can alter the structure of the gut microbiome, further modulating immune cell function, ultimately playing a significant role in the onset and progression of HCC. For example, in 2010, Yu et al. showed that treatment of a mouse model of HCC with a combination of antibiotics (amoxicillin, vancomycin, neomycin sulfate, and metronidazole) depleted host bacteria and thereby impeded progression of HCC. The size and quantity of nodules in the antibiotic treatment group were significantly decreased compared with those in the control group (80). Other researchers have found that the combination of antibiotics (vancomycin, cefoperazone or ampicillin, neomycin, metronidazole, and vancomycin) inducing dysregulation of the gut microbiota in HCC patients can lead to a significant increase in IL-25 concentrations. The underlying mechanism involves dysregulated gut microbiota stimulating colon proliferative epithelial cluster cells to secrete large amounts of IL-25. Subsequently, IL-25 promotes HCC progression by inducing replacement activation of macrophages and the secretion of C-X-C motif chemokine ligand 10 within TME (**Figure 2D**) (81).

Another group utilized a transgenic mouse model of MYC to induce spontaneous HCC and administered an antibiotic mixture containing vancomycin, neomycin, and imipenem + cilastatin in drinking water. They discovered that compared with untreated MYC mice, antibiotic-treated MYC mice exhibited a gradual decrease in HCC incidence. Further investigations revealed that the gut microbiota controlled the accumulation of CXCR6+ liver NKT cells by regulating concentrations of chemokine CXCL16 in hepatic sinus endothelial cells by using BAs as signaling molecules. The accumulated NKT cells exhibited an activated phenotype and produced large amounts of IFN-γ, which effectively inhibited HCC tumor growth (82) (**Figure 2E**).

### 3.2 Probiotics

The Food and Agriculture Organization of the United Nations and the World Health Organization define probiotics as “living microorganisms that, when administered in adequate quantities, confer health benefits on the host” (83, 84). The main types of probiotics include *Bifidobacterium*, *Bacillus subtilis*, and lactic acid bacteria.

A groundbreaking study demonstrated that oral probiotics effectively enhance gut microbiota homeostasis in patients with cancer, thereby improving their response to immunotherapy. Specifically, the use of the probiotic drug CBM588 as an adjuvant significantly enhanced both the median progression-free survival and response rate in patients with metastatic renal cell carcinoma who were treated with nebuliumab, a PD-1 inhibitor, combined with ipilimumab, a CTLA-4 inhibitor. The median progression-free survival was increased by nearly fourfold, and the partial response rate was increased from 20% to 58% (85). The impact of probiotics on tumor immunotherapy has also been demonstrated in melanoma. Specifically speaking, the frequency of interferon-gamma (IFN-γ)-positive CD8+T cells in the probiotic-treated mouse tumors exhibited a significant reduction compared to the control group (86). Although there are no clinical studies on the effect of probiotics on HCC immunotherapy, there have been studies showing that the combination of probiotics and ICIs may also have an impact on HCC treatment.

However, a certain number of studies have been carried out on the effects of probiotics on HCC tumor cells and immune cells in the HCC TME. A randomized clinical trial of surgical intervention in patients with HCC showed that administration of probiotics significantly enhanced intestinal barrier function by increasing the abundance and diversity of beneficial bacteria and inhibiting the proliferation of harmful bacteria (87). It has also been demonstrated that various probiotics, including *L. rhamnosus* GG, *B. longum*, and *Lactobacillus casei*, affecting the incidence and progression of HCC. These probiotics mitigate the risk of HCC through diverse mechanisms, such as modulation of gut microbiota equilibrium, inhibition of HCC cell proliferation, attenuation of inflammatory responses, and regulation of immune function (88). In addition, probiotics can promote epigenetic modification of host genes, thereby reducing the progression of HCC (89).

More interestingly, the administration of probiotic fermented milk was found to attenuate the expression of *rasp-21*, *c-myc*, *cyclin D1*, and *Bcl-2* in rat models of HCC, thereby impeding tumor progression (90). Similarly, Li et al. showed that Pro-hep, a probiotic formulation consisting of *L. rhamnosus* GG, live *Escherichia coli* Nissle 1917, and heat-inactivated VSL#3 (in a ratio of 1:1:1), suppressed HCC development in a murine model. Pro-hep reduced tumor weight and size by 40% in mice. It was determined that Pro-hep attenuated Th17 cell polarization within HCC tumors and downregulated the production of the pro-inflammatory vascular growth factor IL-17, thereby impeding HCC tumor progression (**Figure 2F**). Furthermore, Pro-hep increased the abundance of SCFA-producing bacteria (91).

### 3.3 FMT

FMT is a procedure in which stool from a healthy donor is transferred into the gastrointestinal tract of a recipient. A fecal graft consists of approximately 55% microbes and 24% soluble components, including mucus, fat, protein, small molecules, and SCFAs (92). In 2014, FMT was approved for the treatment of recurrent *Clostridium difficile* infections and demonstrates an approximate effectiveness rate of 90% in eliminating such infections (93). Moreover, FMT has proven efficacious in preventing the recurrence of such infections. The efficacy of FMT in the treatment of liver diseases, such as MASLD and alcoholic hepatitis, has also been demonstrated. For instance, Gomez-Hurtado et al. found that FMT from a healthy donor to 47 patients diagnosed with MASLD led to a significant amelioration of their clinical symptoms. Similarly, in 2017, Philips et al. published a compelling study demonstrating that a patient with severe alcoholic hepatitis exhibited significant clinical and biochemical improvements after undergoing FMT (94).

In addition, the gut microbiota of eight patients with advanced HCC who were treated with nabuliumab exhibited distinct β diversities, In non-responders, bacteria such as pneumococcus, *Escherichia coli, L. regenerans*, *Streptococcus mutans*, *Enterococcus faecium*, *Streptococcus gordonii*, *Veillonella atypica*, *Granulicatella* sp. and *Trichuris trichiura* were prevalent. In contrast, in responders, *Citrobacter freundii*, *Azospirillum* sp., and *Enterococcus durans* were prevalent (95). Furthermore, Ponziani et al. conducted a prospective study on 11 patients with HCC who were treated with tremelimumab and/or durvalumab. Their findings indicate that the relative abundance of *Akkermansia* was higher in responders than non-responders, while the relative abundance of *Enterobacteriaceae* decreases in responders (96). In summary, although the effectiveness of FMT in treating HCC with ICIs has not been clinically proven, the distinct fecal flora composition observed in ICIs responders and non-responders among patients with HCC implies that FMT has the potential to enhance the efficacy of HCC immunotherapy. In addition, there are two clinical trials underway to investigate this (**Table 2**).

**Table 2:** Important clinical trials of FMT combined with immunotherapy for the treatment of HCC.

Start time	Research objective	Method	Phase	Clinical trial ID
2023	Test the safety and efficacy of FMT combined with atezolizumab and bevacizumab in patients with advanced HCC who have not responded to previous immunotherapy.	HCC patients who respond to immunotherapy with PD-(L)1 undergo a single FMT, while HCC patients who fail to achieve complete or partial response to atezolizumab/bevacizumab continue to receive atezolizumab/bevacizumab every 21 days according to the protocol after the single FMT.	II	NCT05750030
2024	Evaluate the safety and immunogenicity of combining FMT with standard care immunotherapy for advanced HCC.	Atezolizumab 1200mg i.v. & bevacizumab 15mg/kg body weight i.v. Oral vancomycin (125 mg 4xd, -3 to 0 days) is used to reduce the gut microbiota of the original patient. FMT was performed on day 0 and day 21 using capsules (50 grams of feces).	II	NCT05690048

However, there is currently one study on the impact of FMT on immune cells in the TME of HCC mice. *Lactobacillus reuteri* is a well-known probiotic that has positive effects on various diseases, such as constipation, MASLD, metabolic syndrome, acute diarrhea, allergic rhinitis, and asthma, and enhances ICIs-based treatment of melanoma (97–100). Recent research showed that a reduction in the abundance of enteroborne *L. reuteri* and in concentrations of its metabolite (acetic acid) are involved in the development of HCC. For example, transplantation of feces treated with antibiotics and supplemented with *L. reuteri* or wild-type mice feces into mice models of HCC (in which the abundance of *L. reuteri* and concentrations of acetic acid were significantly reduced) resulted in a decrease in tumor quantity and size. Acetate produced by *L. reuteri* inhibited interleukin-17A-producing ILC3 cells in HCC by inhibiting histone deacetylase activity and inducing SRY-box transcription factor 13 acetylation (101). Other studies have shown that interleukin-17A can cause MASH and HCC, while interleukin-17A inhibitors can prevent MASH and HCC in high-risk patients (102) (**Figure 2G**). However, FMT has some disadvantages, such as the risk of disease transmission from a donor to a recipient, and variations in patient acceptance (103).

### 3.4 Prebiotics

The term prebiotic is rather recent, as it was first defined in 1995 as “an indigestible food component that selectively stimulates the growth or activity of specific bacteria in the gut to enhance host health” (104). Prebiotics are abundant in various plants, such as onions and garlic, and contribute to the production of SCFAs (105). Currently, fructooligosaccharides and inulin are among the most extensively studied prebiotics. These compounds have demonstrated the ability to promote proliferation of the beneficial bacteria *Bifidobacterium*, which has potential anticancer properties (106).

A robust inverse association was found between the incidence of liver cancer and consumption of vegetables abundant in inulin and fructooligosaccharides, such as celery, mushrooms, and leeks. This association suggests that prebiotics exert a potent protective effect against liver cancer (107). Additionally, a meta-analysis of a total of 3,912 patients diagnosed with HCC demonstrated that each 100-g-per-day increase in vegetable consumption was associated with an 8% reduction in the risk of developing HCC (108). Prebiotics play a crucial role in the prevention of HCC and the mitigation of risks of HCC. However, there is currently insufficient evidence to support the hypothesis that prebiotics enhance immune cell function in the HCC TME or improve HCC immunotherapy by modulating the composition of the gut microbiome. Therefore, further comprehensive studies are needed to verify whether these hypotheses hold true.

### 3.5 Diet

Diet fundamentally shapes the relationship between humans and their gut microbiota, as dietary nutrients profoundly influence the health and viability of trillions of gut microbes (109). Therefore, the effects of diet on gut microbiota may significantly regulate the progression of various diseases.

For instance, it was found in mice that a high-sugar, high-fat diet can lead to changes in the composition of the gut microbiota, resulting in enhanced expression of pro-inflammatory markers TNF-α, IL-2, peroxisome proliferator-activated receptor gamma, and NF-κB within the intestinal tract. These changes triggered tissue inflammation and pathological changes, ultimately leading to sustained inflammation-induced damage in both the small intestine and colon (110). Similarly, it was found that a high-cholesterol diet caused gut microbiota changes in mice and promoted the progression of liver steatosis, hepatitis, liver fibrosis, and HCC. In addition, germ-free mice also showed liver lipid accumulation after ingesting stool samples from the high-cholesterol diet group (111).

Furthermore, prolonged administration of fermentable fiber to TLR5 knockout mice (T5KO) resulted in hyperbilirubinemia and jaundice-associated HCC. Similarly, extensive hepatic inflammation and infiltration of neutrophils, macrophages, and T cells were observed in the livers of mice fed an ICD (an open-source diet containing 7.5% inulin and 2.5% cellulose). Additional investigation revealed that the gut microbiota of these mice exhibited an increased abundance of fibrous fermenters and Proteobacteria, a decreased abundance of Firmicutes, and a reduction in species richness and α-diversity. Moreover, compared with the control group, cecal butyrate concentrations were significantly increased in ICD-fed mice. However, antibiotic-treated T5KO mice showed immunity to ICD-induced HCC, suggesting that the etiology of this form of HCC is microbially dependent, although this remains to be mechanistically confirmed (112). Therefore, despite the numerous advantages of consuming foods rich in soluble fiber, they should be consumed with caution, as they may trigger HCC initiation. However, there is currently a lack of sufficient research on the impact of diet on immune cells in the HCC TME and its potential role in improving immunotherapy. Further in-depth studies in these areas are urgently needed in the future.

### 3.6 Traditional Chinese medicine preparations

Another way to improve the health of the gut microbiota is through the use of traditional Chinese medicine preparations. For example, xiayuxue decoction XYXD (which primarily consists of *Rheum officinale* Baill, *Prunus persica* (L.) Batsch, and *Eupolyphaga sinensis* Walker), was found to trigger the immune action of NKT cells against HCC by regulating the gut microbiota. Its mechanism of action involves increasing the abundance of *Bacteroides* and *Lactobacillus* to promote the production of bile salt hydrolase, which facilitates the conversion of conjugated BAs into primary BAs. Simultaneously, it also reduces the abundance of *Eubacterium* and hinders the conversion of primary BAs into secondary BAs. The increase in the concentrations of primary BAs from both pathways ultimately triggers CXCR6 to recruit NKT cells in the liver, which produce IFN-γ, which has an inhibitory effect toward HCC tumor cells (113) (**Figure 2H**). Other findings also highlight the therapeutic potential of traditional Chinese medicines in regulating gut microbiota to influence cancer progression. For example, Pian Zai Huang can inhibit the development of colorectal cancer by regulating gut microbiota composition and metabolite levels, promoting the improvement of intestinal barrier function, and inhibiting carcinogenic and pro-inflammatory pathways (114).

### 3.7 Bacteriophage

In addition to common methods such as probiotics supplementation and dietary adjustments, phage therapy, as an emerging strategy, opens up a new path for optimizing the gut microbiome. Bacteriophages possess high specificity, excellent manipulability, non-toxicity, and nanometer-scale characteristics, making them highly promising vectors in the fields of targeted therapy and cancer immunotherapy. Currently, cancer research faces numerous challenges, such as damage to healthy cells, suboptimal targeting efficiency, biological barriers, and the emergence of drug resistance. In this context, bacteriophage therapy or the use of bacteriophages as delivery vectors for treatment strategies has become particularly urgent and necessary (115).In addition, as a natural biopreparation, bacteriophages have a relatively short survival time in the human body. Unlike antibiotics, they do not remain in the body for long periods, which results in relatively fewer toxic side effects.

Phage therapy has been studied in diseases such as breast cancer, lung adenocarcinoma, colorectal cancer, hepatocellular carcinoma, B-cell lymphoma, multiple myeloma, cervical cancer, neuroendocrine pancreatic tumors, melanoma, chondrosarcoma, and glioblastoma. Currently, the mechanisms of action of phage-based cancer therapies mainly involve two key aspects. On one hand, phages can trigger the body’s immune response to precisely target and destroy cancer cells. On the other hand, phages can serve as efficient targeted delivery carriers, enabling various therapeutic agents to be directly and accurately delivered to the target cells (115).

It is also worth mentioning that numerous studies are currently focused on using phages to improve the gut microbiota for disease treatment. For example, research by Yuan Jing and colleagues revealed that in NAFLD, some cases of endogenous alcoholic fatty liver disease caused by high ethanol-producing *Klebsiella pneumoniae* were studied using a mouse model. They found that the clinically promising short-tail virulent phage phiW14 could specifically target the high ethanol-producing *Klebsiella pneumoniae*, blocking the production of endogenous ethanol, regulating immune responses and metabolism, and alleviating liver damage, providing an efficient and specific therapeutic approach for endogenous alcoholic fatty liver disease without affecting the gut microbiota structure (116).

Another study indicates that in colorectal cancer (CRC) patients, the number of *Fusobacterium nucleatum* is increased in feces and enriched at the tumor site, while butyrate-producing bacteria such as *Faecalibacterium prausnitzii* are reduced. *Fusobacterium nucleatum* helps CRC cells resist chemotherapy through the TLR4-Myd88 pathway, while butyrate secreted by *Faecalibacterium prausnitzii* inhibits CRC cell growth. The study also found that *Fusobacterium nucleatum*-specific phages isolated from human saliva, combined with drug-loaded nanoparticles, formed a nanomedicine that was safe in piglets. In mice, the nanomedicine was able to accumulate at CRC tumor sites, significantly extend the survival of CRC mice, reduce intestinal adenomas in Apc mice, and also decrease the number of *Fusobacterium nucleatum*, increase the abundance of *Faecalibacterium prausnitzii*, and elevate butyrate levels in the colon (117).

Although there is currently no research specifically exploring the use of phages to optimize the gut microbiota to improve immune cell function in the HCC TME or advance immunotherapy, existing studies have provided valuable insights. In the future, phages are expected to become a key intervention in HCC treatment. For example, research can analyze the interaction between phages and the gut microbiota at the molecular and cellular levels, use high-throughput screening to identify effective strains, and develop personalized treatment strategies based on multi-omics technologies. By combining basic and clinical trials, exploring the combination of phages with other therapeutic approaches may optimize treatment plans, improve patient prognosis, and open up new pathways for clinical HCC treatment.

## 4 Summary and prospects

In conclusion, this review provides an in-depth analysis of the multiple mechanisms through which the gut microbiota influences the development of HCC. On one hand, the gut microbiota contributes to the progression of HCC through various pathways, such as affecting liver health, exerting its metabolites on HCC tumor cells, and modulating immune cell functions. This offers a new perspective for researchers to explore the pathogenesis of HCC. On the other hand, studies have shown a close relationship between the gut microbiota and the efficacy of HCC immunotherapy, with compositional differences significantly impacting patients’ responses to ICIs. This finding is crucial for identifying key factors influencing the effectiveness of immunotherapy and provides a solid theoretical foundation for optimizing treatment strategies.

From a practical standpoint, the findings of this review hold significant value in multiple aspects. For example, in terms of treatment optimization, interventions based on gut microbiota research—such as the rational use of antibiotics, supplementation with probiotics and prebiotics, FMT, and traditional Chinese medicine preparations—can help regulate the gut microbiota, enhance the effectiveness of immunotherapy, and inhibit HCC progression, thus providing viable methods to improve patient survival rates and quality of life. In the field of personalized medicine, the use of gut microbiota sequencing and multi-omics technologies can precisely predict patients’ responses to various treatments, enabling the development of personalized treatment strategies tailored to each patient, ultimately achieving precision medicine and significantly enhancing treatment targeting and effectiveness. In terms of disease prevention, clarifying the relationship between diet, specific nutrients (such as dietary fiber and prebiotics), and HCC risk can help guide the public in adjusting their diets and lifestyles to reduce the risk of liver cancer. Furthermore, the correlation between the status of the gut microbiota and the prognosis of HCC patients suggests that the microbiota could become an effective prognostic indicator, assisting clinicians in more accurately assessing disease progression and making more scientifically informed treatment decisions.

Although the role of gut microbiota in influencing HCC immune cells and immunotherapy is not yet fully understood, current research primarily focuses on exploring the correlation between gut microbiota and HCC. Some studies may have deficiencies in experimental design, such as the absence of a control group or inadequate consideration of other confounding factors, which could affect the interpretation of their results. More importantly, the composition of the gut microbiota varies significantly across populations, influenced by geographic and ethnic differences. However, the gut microbiota holds tremendous potential and promises that warrant attention from researchers. In the future, innovations such as synthetic biology and engineered probiotics may offer new approaches for therapeutic modulation of gut microbiota. Additionally, the integration of artificial intelligence and machine learning with microbiome data could revolutionize our ability to predict and optimize personalized microbiome-based treatments. However, translating research findings into practical treatments remains a formidable challenge, requiring interdisciplinary collaboration and extensive clinical research to ensure their efficacy and safety.

In conclusion, future research efforts could focus on elucidating the molecular mechanisms by which gut microbiota influence HCC and exploring microbiota-targeted therapies. The integration of microbiome sequencing technology, personalized medicine models, and novel biotechnological advances holds great promise in revolutionizing HCC treatment, significantly improving patient outcomes, and bringing new hope to HCC patients.
